# Tunable daytime passive radiative cooling based on a broadband angle selective low-pass filter†

**DOI:** 10.1039/c9na00557a

**Published:** 2019-11-04

**Authors:** Nelson W. Pech-May, Markus Retsch

**Affiliations:** Department of Chemistry, University of Bayreuth Universitätsstr. 30 95447 Bayreuth Germany nelson.pech@uni-bayreuth.de markus.retsch@uni-bayreuth.de

## Abstract

Passive daytime cooling could contribute to the reduction of our global energy consumption. It is capable of cooling materials down to below ambient temperatures without the necessity of any additional input energy. Yet, current devices and concepts all lack the possibility to switch the cooling properties on and off. Here, we introduce dynamic control for passive radiative cooling during daytime. Using an angle-selective solar filter on top of a nocturnal passive radiator allows tuning the surface temperature of the latter in a wide range by just tilting the filter from normal incidence up to around 23°. This angle-selective filter is based on optically engineered, one-dimensional photonic crystal structures. We use numerical simulations to investigate the feasibility of a switchable low-pass filter/emitter device.

## Abstract 2

Dynamic control for passive radiative cooling during daytime.

## Graphical abstract

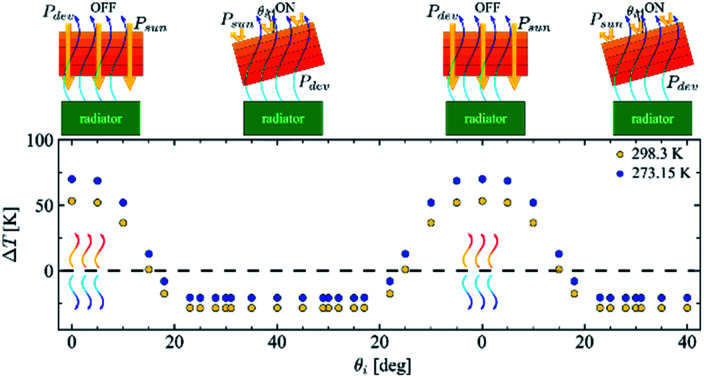

## Introduction

Daytime passive radiative cooling has recently gained attention due to its high potential for cooling surfaces even when exposed to solar radiation.^1–5^ Ideally, this technology can cool surfaces even below the ambient temperature without any external power input. Furthermore, nocturnal passive radiative coolers, which have been studied since the 1970s, can only produce net cooling during night, when the cooling material is not exposed to solar radiation.^6,7^ The physical mechanism responsible for passive radiative cooling is based on the exchange of heat by radiation between a surface or body (at around 300 K) on the surface of the earth and outer space, which is cold (at around 2.8 K). To exchange heat by radiation, this surface must show high emissivity within the sky-window: 7.5 ≤ *λ* ≤ 14 μm.^1^ The reason is that this spectral range coincides with one of the transparent bands of the atmosphere. Moreover, the blackbody radiation of a surface at a temperature of ∼300 K peaks in the same spectral range. Briefly, a surface with high emissivity within the sky-window will passively cool itself by radiative heat transfer to the cold outer space. This principle is applied for cooling during night. However, during daytime, solar radiation must be reflected or scattered effectively to cool down a surface.

Various approaches have been proposed to obtain daytime passive radiative coolers, including photonic structures,^4^ microparticles embedded in a polymer,^5^ hierarchically porous polymers,^3^ complete delignification and densification of wood,^8^ among others. Nevertheless, all of these very different approaches are only able to produce fixed amounts of cooling power, unchangeable once their intrinsic characteristics are determined by the materials design. In practice, it would be desirable to control the cooling (or heating) power of a device at will, such as in a fridge or an air-conditioner. Additionally, depending on the year's season, one would need heating (in winter) or cooling (in summer) power to maintain a comfortable room temperature.

One way to tune the cooling power during daytime consists in gaining dynamic control over the reflection of the solar spectrum. In this way, one would account for the amount of radiated power from the sun that could be absorbed by the radiator surface. It was only until 2014, when the group of J. Joannopoulos demonstrated a broadband reflector with angular selectivity.^9^

Angle selectivity in 1D photonic crystals is based on the zero reflection occurring at the interface between two media with p-polarized light at the Brewster angle, which is usually larger than 45°.^9,10^ This angle is specific for each wavelength. To achieve broadband applicability, multiple periodicity in the 1D stratified structure is needed.^9,11^ Up to now, research has been focused on studying angle-selective broadband filters in the visible range, considering mainly p-polarized light. Nevertheless, the design of an angle-selective solar filter must consider both polarization states (s-polarized and p-polarized light). This is critical because solar radiation is mostly unpolarized. To fulfill this condition, the filter components should be impedance-matched with the surrounding air.^9^ Moreover, another advantage of impedance-matching with air is that in this case, the Brewster angle occurs at normal incidence. Here, we have combined both approaches: impedance-matching^9^ for angle selectivity around normal incidence (valid for both polarization states) and broadband reflectivity^11^ from 1D stratified stacks of multiple periodicity, to successfully design an angle-selective solar filter.

In this work, we propose an angle-selective solar filter on top of a nocturnal passive radiative surface, to achieve tunable radiative cooling (or heating) power during daytime. The impedance-matched filter serves as an active control of the reflected solar radiation, while the nocturnal radiator cools itself by thermal emission through the sky-window. The components of the 1D stratified structure need to be transparent in the sky-window range to allow transmission of the radiator. Numerical simulations performed considering 298.3 K atmospheric temperature show that the radiator surface can be tuned from 270 K to 352 K by just tilting the solar filter from normal incidence (0°) up to around 23°. The designed filter consists of 75 quarter-wave stacks of geometrically increasing periodicity, each one composed of 64 unit-cells or bilayers. The characteristics of the filter have been tailored using the transfer-matrix method to obtain the transmittance spectra.^12,13^ On the other hand, the nocturnal radiator consists of a microstructured silica photonic crystal which is also a solar absorber.^2^ This radiator has been chosen to prove the cooling (or heating) tunability enabled by the proposed filter-radiator device. Moreover, our results show that controlling the reflection of solar radiation with a filter, instead of the static approach in daytime passive radiative cooling, structuring the radiator surface itself to reflect or scatter solar radiation paves the way to achieve tunable passive radiative cooling during daytime.

## Concept of an angle-selective filter for the solar spectrum

We introduce a 1D photonic device to model a broadband filter that covers the whole solar spectrum (0.26 μm ≤ *λ* ≤ 2.5 μm). The aim of this filter is to transmit electromagnetic waves with wavelengths longer than 2.5 μm and to reflect all incoming waves with wavelengths within the solar spectrum. Moreover, this filter operates as described for any state of polarization of the incoming waves and above a given angle of incidence.

**Fig. 1:**
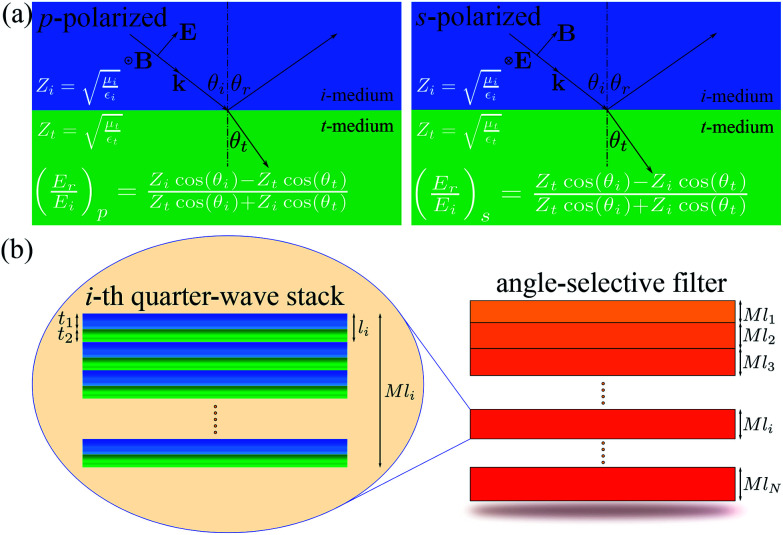
(a) Diagram of p-polarized and s-polarized light at the interface of two media. (b) Geometry of a broadband angle selective filter based on *N* quarter-wave stacks of increasing thickness. Each quarter-wave stack is conformed by *M* bilayers satisfying the Bragg condition.

Consider an incidence medium (i-medium) of refractive index 
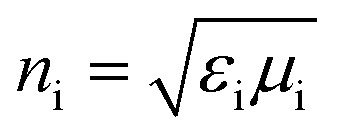
 in contact with a transmittance medium (t-medium) of refractive index 
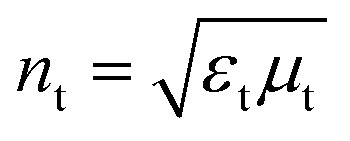
,where *ε*_x_ is the electric permittivity and *μ*_x_ is the magnetic permeability of each medium x = {i, t}. Fig. 1a shows the reflectivity equations, *i.e.*, the ratio between the magnitudes of the reflected electric field (*E*_r_) and the incident electric field (*E*_i_), for both s- and p-polarized incident light. The incident electromagnetic (EM) waves, with electric field *E* and magnetic field *B*, have wavevector *k* with incidence angle *θ*_i_. The direction of the reflected and transmitted EM waves is characterized by the reflectance angle (*θ*_r_ = *θ*_i_) and transmittance angle (*θ*_t_), respectively. The reflection fields at the boundary between these two media are given by the well-known Fresnel equations.^14^ These expressions show that the impedance of each medium governs the reflectivity. In particular, for impedance-matched media (*Z*_i_ = *Z*_t_), reflection becomes zero at both polarization states because cos *θ*_i_ = cos *θ*_t_. This is satisfied when the incidence angle *θ*_i_ equals the transmittance angle *θ*_t_. Therefore, the zero reflection condition at the boundary of two impedance-matched media with different refractive indices is only satisfied for normal incidence *θ*_i_ = *θ*_t_ = 0, as can be inferred from Snell's law (*n*_i_ sin *θ*_i_ = *n*_t_ sin *θ*_t_).

It has been shown in the literature that reflection over a broadband frequency range can be obtained by piling up two or more 1D photonic crystals of appropriate periodicities.^10,11^ Accordingly, a combination of the impedance-matched condition and multiple periodicity quarter-wave stacks allows us to design broadband filters which transmit electromagnetic (EM) waves at normal incidence and reflect all EM waves of larger incidence angles.

The geometry of the proposed solar filter is shown in Fig. 1b. It is composed of *N* Bragg sub-filters of different periodicities (*l*_1_ ≤ *l*_*i*_ ≤ *l*_*N*_). Each quarter-wave stack consist of *M* bilayers (unit cells) with defined periodicity according to Bragg's diffraction condition, *i.e.*, the thickness of each layer times its refractive index equals one quarter of the diffracted wavelength. Consequently, the width of the principal stop-band of each stack depends on the ratio of the refractive indices between the two components and is proportional to the diffraction frequency.^15,16^ As an example, the *i*-th quarter-wave stack is zoomed in Fig. 1b. Its thickness is *Ml*_*i*_, where *l*_*i*_ = *t*_1_ + *t*_2_ is the period of the *i*-th unit cell. The thicknesses of layer 1 (in blue) and layer 2 (in green) are *t*_1_ and *t*_2_, respectively. The thickness of the *i*-th period is defined by *l*_*i*_ = *l*_0_*r*^*i*−1^, where *l*_1_ = *l*_0_ is the starting period of the filter and *r* = *t*_2_/*t*_1_ is the ratio between the thicknesses of the two layers forming the unit cell and is constant for all stacks of the filter. It has been shown in the literature that increasing the periodicity using a geometrical progression provides comparable results as choosing each periodicity from a nonlinear optimization algorithm for a given broadband spectrum.^9^

Using the presented concept, we model a filter such that layer 1 is impedance-matched with layer 2 and with the surrounding air (*Z*_1_ = *Z*_2_ = *Z*_0_). Accordingly, we set *ε*_1_ = *μ*_1_ = 1 and *ε*_2_ = *μ*_2_ = 2 for layers 1 and 2, respectively. In all cases, the transmittance has been computed using the transfer-matrix method (TMM).^12,13^

### Tailoring the solar spectrum

**Fig. 2:**
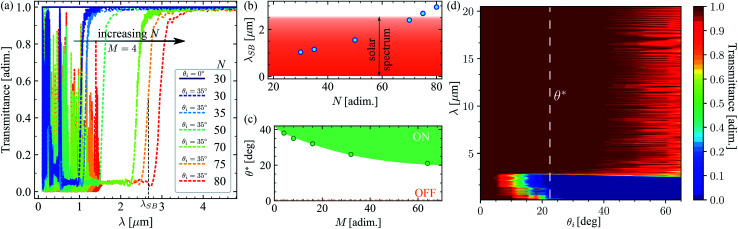
(a) Exemplary transmittance spectra for different numbers of quarter-wave stacks at a constant number of unit cells (*M* = 4). (b) Shift of the stop-band wavelength as a function of the number of quarter-wave stacks *N*. (c) Dependence of the optimum operation angle θ* of the filter on the number of unit cells for *N* = 75. (d) Chart of the transmittance spectra for an angle selective solar filter with *N* = 75 and *M* = 64. The dashed vertical line indicates the operation angle of this filter. Note that the transmittance is zero in the whole solar spectrum and sharply increases to its maximum value for *λ* > 2.6 μm.

Fig. 2a shows the effect of increasing the total number of stacks *N* in the filter. The larger the number of stacks, the longer the stop-band wavelength *λ*_SB_ of the filter. We have defined *λ*_SB_ as the wavelength at which the transmittance of the filter equals 0.5 when the filter is active. The filter is said to be active or ON when it does not transmit any light up to its stop-band wavelength, but transmits all light above this wavelength. In contrast, the filter is OFF when it transmits all light at any wavelength, as shown in Fig. 2a by the dark blue spectrum (*θ*_i_ = 0°). Due to the fact that *λ*_SB_ is almost independent of the incidence angle when the filter is ON (as confirmed in Fig. 2d), any particular selection of *θ*_i_, which makes the filter ON, is valid to explore the effect of increasing the number of quarter-wave stacks (*N*) for shifting *λ*_SB_ to longer wavelengths.

Accordingly, in Fig. 2a, we have fixed the incidence angle to 35°, to explore the shift of the stop-band wavelength for 30 ≤ *N* ≤ 80. Even though this is not the optimum angle for operation of these filters, it is a good choice to guarantee that the filters are active, as shown by their corresponding spectra. The stop-band wavelength as a function of the total number of stacks for *M* = 4 unit-cells in each stack, *r* = 1.02 and *l*_0_ = 200 nm, is shown in Fig. 2b. It shows a nonlinear dependency on *N*, because the stop-band frequency of each stack is proportional to its mid-gap frequency. This changes according to the previously defined geometrical progression (*l*_*i*_ = *l*_0_*r*^*i*−1^).^11,15^ To cover the entire solar spectrum, we have chosen *N* = 75 stacks for the filter, as indicated in Fig. 2b.

### Tailoring the angle-selectivity

How many unit-cells should be included in each of the *N* Bragg-stacks? For this, we explore the effect of the number of unit cells *M* on the transmittance spectrum of a filter with *N* = 75, *r* = 1.02 and *l*_0_ = 200 nm, which has been optimized for the solar spectrum. We define the optimum operation angle θ* as the minimum incidence angle for which transmittance is zero in the solar spectrum, but all radiation is transmitted for wavelengths larger than *λ*_SB_ = 2.6 μm. Fig. 2c shows that increasing the number of unit cells *M* in each stack reduces θ*. This is in agreement with the fact that the reflectivity of a 1D photonic crystal reaches the ideal value of 1 when increasing the number of unit cells or when increasing the incidence angle.^10,17^ Naturally, in this case, increasing the number of unit cells means that the maximum reflectivity (zero transmittance) can be achieved at smaller incidence angles. Accordingly, an optimum incidence angle θ* = 23° was obtained for *M* = 64. This is indicated in Fig. 2d with a vertical dashed line.

Fig. 2d shows that for normal incidence (*θ*_i_ = 0°) all radiation is transmitted in the spectrum 0.1 ≤ *λ* ≤ 20.5 μm and the filter is OFF. The OFF state is indicated by the dashed line in Fig. 2c. For incidence angles 0 < *θ*_i_ < θ*, the filter is ON, but only larger wavelengths within the solar spectrum are reflected, while all other radiation is transmitted. This is because the edges of the band gaps for 1D photonic crystals shift to higher frequencies (short wavelengths) when increasing the incidence angle.^11,18^ On the other hand, for incidence angles *θ*_i_ > θ*, the filter is ON and the entire spectrum of solar radiation is reflected. In this case, all radiation with a wavelength larger than *λ*_SB_ = 2.6 μm is transmitted. Nevertheless, for *θ*_i_ ≳ 40°, the presence of interference reduces the total transmitted radiation. For this reason, the optimum performance of the filter is obtained for incidence angles θ* ≤ *θ*_i_ ≲ 40°. In this range of incidence angles, the whole solar radiation is fully reflected and all radiation within the sky-window (7.5 ≤ *λ* ≤ 14 μm) is completely transmitted. It is worth pointing out that θ* does not mark a critical angle, but instead a threshold angle for the optimum operation of the filter. More details about the designed filter are provided in the ESI.†

Such a filter is challenging to construct in the laboratory. On the one hand, layer 1 needs a refractive index close to that of air (*n*_1_ = 1) over the whole wavelength range. Recently, nano and microstructured photonic materials have shown to be appropriate to meet the optical properties required for impedance-matching to air in a broadband spectrum. In particular, 3D thin-shell nanolattices made out of Al_2_O_3_ or ZnO have shown refractive indices around of 1.025 in a broadband spectrum.^19^ Additionally, arrays of SiO_2_ nanorods have shown low refractive indices down to 1.08. Similarly, phase separation of nanoporous thin polymer films (PMMA-PS) has also shown low refractive indices down to 1.05.^20,21^ On the other hand, layer 2 requires a refractive index *n*_2_ = 2 over the same wavelength range. This could be realized by nanocomposites comprising high refractive index polymers, such as recently discovered sulfur-based polymers,^22^ and high refractive index inorganic nanoparticles such as boron nitride or titania. A further difficulty is the necessity to fabricate such promising refractive index optimized structures into thin films of high quality.

## Energy balance of a filter-radiative cooler system

**Fig. 3:**
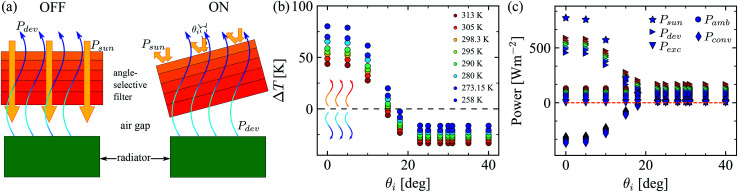
(a) Diagram of the proposed radiator-filter device. Under normal incidence, the solar filter is OFF, but it is turned ON by tilting it. The radiator is able to cool itself when the filter reflects all the solar radiation. (b) Temperature difference (Δ*T* = *T* − *T*_amb_) between the radiator surface and the ambient as a function of the incidence angle. Positive values mean that the radiator is being heated, while negative values refer to the cooling of the radiator surface. (c) Computed power contributions per unit area for the radiator, see eqn (1), as a function of the incidence angle. The maximum net cooling is obtained for *θ*_i_ ≥ 23° and is around 140 Wm^−2^. All calculations are performed assuming steady-state conditions.

We now investigate the integration of such an angle-selective filter into a passive daytime cooling device. We propose an angle-selective solar filter on top of a nocturnal radiative cooler (radiator), as shown in Fig. 3a. The radiator consists of a photonic solar absorber covered by a transparent thermal blackbody and its spectral emissivity is plotted in the ESI.† The air gap between the nocturnal radiator and the filter is larger than the wavelengths of the incident radiation, *i.e.*, much larger than 20 μm. The operation principle of this device is controlled by the filter: when the filter is OFF (normal incidence), radiative cooling is overwhelmed by solar radiation heating. Therefore, the radiator will be heated up, since its emissivity (absorbance) is non-zero in the solar spectrum. On the other hand, when the filter is at its optimum operation angle (*θ*_i_ = θ*), maximum cooling is obtained from this device. The power balance between the filter-radiator system can be expressed as a pair of coupled integro-differential equations:^23^

1 *P*_total,rad_ = *P*_sun_ + *P*_atm_(*T*_amb_) + *P*_conv_(*T*,*T*_fil_) + *P*_exc_(*T*,*T*_fil_) − *P*_dev_(*T*),

2 *P*_total,fil_ = *P*_sun,fil_ + *P*_atm,fil_(*T*_amb_) + *P*_conv,fil_(*T*,*T*_fil_,*T*_amb_) − *P*_exc_(*T*,*T*_fil_) − *P*_dev_(*T*) − *P*_fil_(*T*_fil_),

where the total cooling power per unit area of the radiator is given by *P*_total,rad_ = *ρ*_rad_*c*_rad_/*A*_rad_(d*T*/d*t*) and the total cooling power per unit area of the filter is *P*_total,fil_ = *ρ*_fil_*c*_fil_/*A*_fil_(d*T*_fil_/d*t*). The density and specific heat capacity of each component are represented by *ρ*_x_ and *c*_x_, respectively. Similarly, *A*_x_ stands for the surface area of each component under normal incidence of solar radiation. The sub-index x = {rad, fil} labels each component. *T*_amb_ is the ambient temperature of the surroundings.

A radiative cooling system as the one in Fig. 3a can only supply sensible cooling if *P*_dev_(*T* = *T*_amb_) exceeds all incoming heating at the initial *T*_amb_ (see eqn (1)). In this case, as time passes, the radiator temperature drops below the ambient temperature, down to a steady state temperature *T*.^1,6^ When the steady state is reached, the filter-radiator system is in thermal equilibrium with the surroundings. Therefore, the total energy of both the radiator and the filter must remain constant. This implies that the total cooling power (derivative of the energy with respect to time) of the radiator and the filter goes to zero, *i.e.*, *P*_total,rad_ = *P*_total,fil_ = 0. Consequently, the system of eqn (1) and (2) simplifies into a pair of simultaneous homogeneous equations.^24^ This system can be solved numerically to obtain the temperature of the radiator (*T*) and the temperature of the filter (*T*_fil_).

The total cooling power of the radiator presented in eqn (1) is equal to the sum of the absorbed power from the solar radiation *P*_sun_, the radiatively absorbed power from the surrounding atmosphere *P*_atm_(*T*_amb_), the convective power between the air gap and the (upper) surface of the radiator *P*_conv_(*T*,*T*_fil_), the radiative power exchanged from the bottom surface of the filter to the surface of the radiator *P*_exc_(*T*,*T*_fil_) and the negative of the power emitted by the device *P*_dev_(*T*). This is the power emitted by the combined radiator-filter system to outer space through the atmospheric windows, when the radiator surface is at temperature *T*. Accordingly, *P*_dev_(*T*) is the actual cooling power of the radiator surface in the presence of the filter. These radiative powers have been computed taking into account the presence of the filter on top of the radiator.

Similarly, the total cooling power of the filter given in eqn (2) is equal to the sum of the absorbed power from the solar radiation *P*_sun,fil_, the radiatively absorbed power from the surrounding atmosphere *P*_atm,fil_(*T*_amb_), the convective power from the surrounding air to the upper surface of the filter and between the air gap and the bottom surface of the filter *P*_conv,fil_(*T*,*T*_fil_,*T*_amb_), the negative of the radiative power exchanged from the bottom surface of the filter to the surface of the radiator *P*_exc_(*T*,*T*_fil_), the negative of the intrinsic power emitted by the device *P*_dev_(*T*) and the negative of the power emitted by the upper surface of the filter *P*_fil_(*T*_fil_).

From eqn (1), *P*_sun_ is the input power per unit area from the sun to the radiator screened by the filter. This means that the emissivity of the combined filter-radiator is used: 
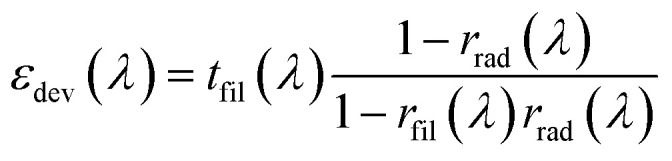
, with *r*_rad_(*λ*) = 1 − *ε*_rad_(*λ*). Accordingly, 
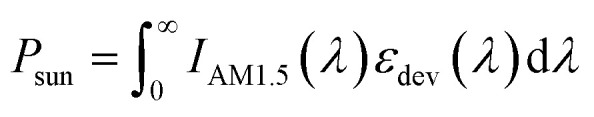
, where *I*_AM1.5_(*λ*) is the spectral irradiance of the sun.^25^*ε*_rad_(*λ*) stands for the angle averaged emissivity of the radiator. This assumption is valid, since it is well-known that the angle dependency of the emissivity of a nocturnal radiator surface slightly decreases for large incidence angles (*θ*_i_ > 70°).^2,7^ On the other hand, the emissivity of the device depends on the incidence angle, because the transmittance of the filter is a function of the incidence angle *θ*_i_. Consequently, *P*_sun_ is also a function of the incidence angle.

The input power from the atmosphere to the radiator in the presence of the filter is 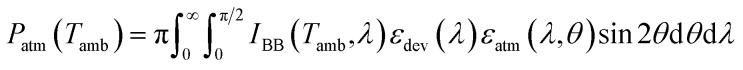, where the emissivity of the atmosphere is computed as *ε*_atm_(*λ*,*θ*) = 1 − [1 − *t*_atm_(*λ*,0°)]^1/cos*θ*^ and *t*_atm_(*λ*,0°) is the transmittance spectrum of the atmosphere at zenith.^26^*I*_BB_(*T*,*λ*) represents the blackbody spectral irradiance at the specified temperature, according to Planck's law.

The convective loss in the upper radiator surface due to the air gap between the radiator and the filter is *P*_conv_(*T*,*T*_fil_) = *h*_conv_[(*T* + *T*_fil_)/2 − *T*], where *h*_conv_ is the heat transfer coefficient due to convection to the surrounding air. The air gap temperature is considered as the mean value between the temperature of the radiator *T* and the temperature of the filter *T*_fil_. A typical value of the heat transfer coefficient due to natural convection (*h*_conv_ = 8 Wm^−2^ K^−1^) has been used for all calculations.^1^

Heat conduction to the surrounding air can be neglected in the limit of low humidity in the atmosphere. However, for situations in which this approximation is not fulfilled (high humidity), eqn (1) and (2) turn into a pair of coupled conductive-convective-radiative integro-differential equations.^27^

The exchanged power by radiation between the radiator surface and the bottom surface of the filter is 
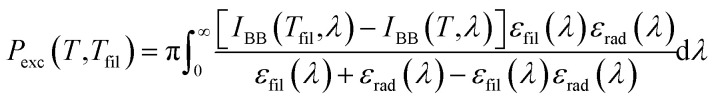
, where *ε*_rad_(*λ*) and *ε*_fil_(*λ*) are the spectral emissivities of the radiator and the filter, respectively.

The cooling power of the device, *i.e.*, the radiator in the presence of the filter, is given by 
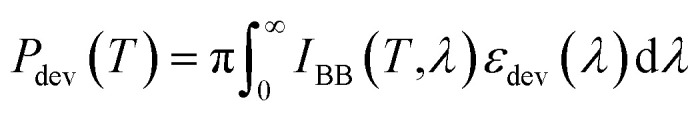
. The corresponding power expressions for the filter are provided in the ESI.†

## Tunable daytime passive radiative cooling

The filter shown in Fig. 3a is an angle-selective solar filter with *N* = 75 and *M* = 64, which was studied above. The radiator is a solar absorber covered by a visibly transparent thermal blackbody, based on a silica photonic crystal.^2^ We have chosen such a radiator to prove that it is possible to achieve tunable daytime passive radiative cooling with the proposed filter-radiator device. Other radiator configurations with distinct spectral properties are given in the ESI (Fig. S4†). The operation principle of the radiator-filter device is that heating or cooling can be tuned by simply tilting the angle-selective solar filter. Nevertheless, the angular location of the sun must be considered when selecting the appropriate tilting angle of the filter. This is because the incidence angle (*θ*_i_) is the relative angle between the solar radiation and the normal to the surface of the filter.

Fig. 3b shows the temperature difference between the radiator surface and the ambient (Δ*T* = *T* − *T*_amb_) as a function of the incidence angle *θ*_i_. Eight different values of ambient temperature (color coded) have been studied to cover a wide range of typical situations. All calculations assume steady-state conditions.

The temperature difference Δ*T* is positive at incidence angles lower than 15°. This means that the radiator is being heated because all (normal incidence) or part of the solar radiation reaches the radiator surface. In this case, the cooling power emitted through the sky-window is not enough to overcome the heating induced by the incident solar radiation. On the other hand, for larger incidence angles (*θ*_i_ > 15°), Δ*T* is zero or negative, which indicates that the radiator can cool itself by emitting radiation through the sky-window, down to temperatures equal to or lower than the ambient temperature. Due to the fact that the filter can only reflect the entire spectrum of solar radiation for incidence angles *θ*_i_ ≤ θ*, the lowest temperature this radiator can reach is obtained for incidence angles larger than or equal to 23°. The lowest radiator temperature achieved with this device is around 270 K and the maximum temperature is 351.6 K. Accordingly, we have shown that it is possible to tune between heating and cooling (or *vice versa*) of the radiator in a wide range of temperatures by changing the incidence angle between solar radiation and the filter.

Fig. 3c shows the power contributions per unit area for the nocturnal radiator as a function of the incidence angle. Each contribution appearing in eqn (1) is represented. Results for the eight different ambient temperatures studied are displayed in Fig. 3b (same color code). The cooling power of the radiator (right triangles) obtained for *θ*_i_ ≥ 23° is around 140 Wm^−2^.^1,28^ This gives the lowest temperature of the radiator.

The incident radiation from the sun to the radiator (star markers) is reduced from 772.6 Wm^−2^ down to around 7 Wm^−2^ by changing the incidence angle from normal incidence to *θ*_i_ ≥ 23°, respectively. This reduction is independent of the ambient temperature, as expected. The incident power from the atmosphere to the radiator (dot markers) is almost independent of the incidence angle and only changes slightly with the ambient temperature. This is because the emissivity of the atmosphere only shows significant changes for incidence angles larger than 60°. The radiative exchange power between the filter and radiator surfaces (downward triangles) is zero for all incidence angles, which agrees with the steady-state condition. The convective power (diamond markers) between the air gap and radiator changes from a cooling mechanism (*T* > *T*_amb_) to a heating source (*T* < *T*_amb_) for the radiator surface. The sum of terms on the right hand side of eqn (1) is represented by the dashed line. *P*_total,rad_ = 0 for any incidence angle, as expected in the steady-state regime. More details are given in the ESI.†

## Conclusion

In this work we have shown that tunable daytime passive radiative cooling is possible by using an angle-selective solar filter on top of a nocturnal radiative cooler surface. We have provided guidelines for the design of such an angle-selective solar filter, based on a one-dimensional photonic crystal composed of multiple Bragg-stacks with different periodicities designed to cover the entire solar spectrum. We have shown that the proposed device operates at its maximum performance for incidence angles equal to or larger than 23°. The surface temperature of the radiator can be controlled from 270 K to 352 K in an atmosphere of 298.3 K, with a maximum net cooling power of ∼140 Wm^−2^. This is comparable to the optimum performance of static devices. Our findings show that the proposed filter-radiator concept is promising for active control of daytime passive radiative cooling.

## Funding sources

This project has received funding from the European Research Council (ERC) under the European Union's Horizon 2020 research and innovation program (grant agreement No. 714968).

## Conflicts of interest

There are no conflicts to declare.

## Supplementary Material

NA-002-C9NA00557A-s001
