# Supplementary material for: Tunable daytime passive radiative cooling based on a broadband angle selective low-pass filter
Source: Nanoscale Adv. 2019 Nov 4;2(1):249–55. doi: 10.1039/c9na00557a (PMC9419168; doi:10.1039/c9na00557a)
Supplement: NA-002-C9NA00557A-s001 [file NA-002-C9NA00557A-s001.pdf]

Electronic Supplementary Material (ESI) for Nanoscale Advances.
This journal is © The Royal Society of Chemistry 2019

Supplementary Information: Tunable daytime passive radiative cooling based on a broadband angle selective low-pass filter

Nelson W. Pech-May[1,*] and Markus Retsch[1,†]

[1] *Department of Chemistry, University of Bayreuth, Universitätsstr. 30, 95447 Bayreuth, Germany*

(Dated: October 9, 2019)

CONTENTS

DETAILS ABOUT THE FILTER 1
Geometrical details 1
Transmittance as a function of angle 1

COMPLEMENTARY POWER CALCULATIONS 2
Energy balance for the radiator and filter 2
Power expressions for the filter 2
Ambient temperature performance 3

References 5

DETAILS ABOUT THE FILTER

Geometrical details

Following the geometric progression presented in the main text ($l_i = l_0 r^{i-1}$), we plot in Fig. S1 the change in the period of each stack in the filter as a function of the i-th stack. Because the nonlinear change in periodicity, the stop-band wavelength is expected to approach non-linearly (when increasing N) to 2.5 μm, as discussed in the main text.

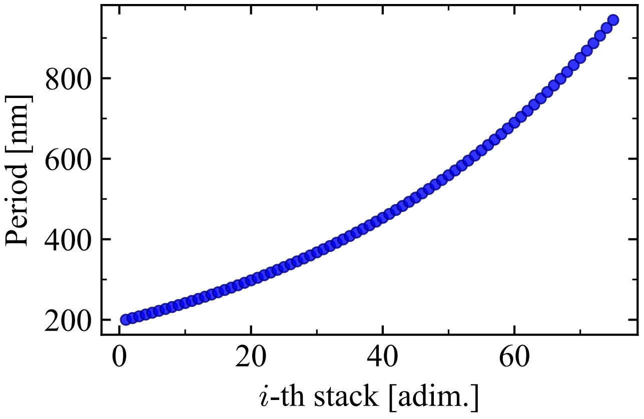

FIG. S1. Period as a function of the i-th stack in the multi-layered filter.

Moreover, the period equals to the sum of the thickness of layer 1 (t_1) and thickness of layer 2 (t_2). The corresponding values of these thicknesses are listed in Table S1, which can be used as guidelines for the construction of such a multilayered filter.

The area covered by the filter (A_f) should be slightly larger than that of the radiator (A_c). This ensures that the later is fully covered when the filter is tilted. For a tilting angle of 20° the ratio of areas is $A_c/A_f \approx 0.94$.

Transmittance as a function of angle

An analytical expression for the reflectance of a non absorbing multilayer Bragg-filter was given by Yeh *et al.*[1] From this expression we can explore the dependence on both the number of unit-cells M in the filter and on the incidence angle θ_i. Accordingly,

$$R_{fil} \approx 1 - \frac{4\exp[-2(M-1)K_i l_0]}{\left(\frac{\sqrt{n_2^2-\sin^2\theta_i}}{\sqrt{n_1^2-\sin^2\theta_i}} + \frac{\sqrt{n_1^2-\sin^2\theta_i}}{\sqrt{n_2^2-\sin^2\theta_i}}\right)^2 \sin^2(k_{2x}t_2)}, \quad \text{(S1)}$$

where K_i is the imaginary part of the Bloch wavenumber, l_0 is the period of the multilayered filter, n_1 and n_2 are the refractive indices for layers 1 and 2, respectively. The thickness of the second layer is t_2 and k_{2x} is the wavevector component parallel to the interface between layers 1 and 2. For non absorbing filters, $T_{fil} = 1 - R_{fil}$.

Equation S1 shows that reflectivity approaches to 1, at a given incidence angle, when increasing the number of unit-cells, because of the exponential term in the numerator. On the other hand, reflectivity will approach 1 (for a given M), when increasing the incidence angle, because the denominator is an increasing function of θ_i. This explains the reduction in the optimum operation angle θ^* when increasing M, in Fig. 2c of the main text.

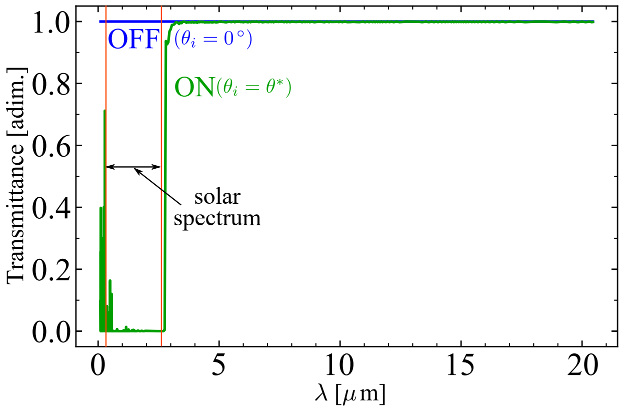

FIG. S2. Transmittance spectra for normal incidence (blue, OFF-state) and for $\theta_i = \theta^*$ (green, ON-state).

* nelson.pech@uni-bayreuth.de
† markus.retsch@uni-bayreuth.de

TABLE S1. Relation of the corresponding thicknesses of layers 1 (t_1) and 2 (t_2) for a filter with $N = 75$ stacks.

i	t_1 [nm]	t_2 [nm]	i	t_1 [nm]	t_2 [nm]
1	99	101	39	220	224
2	101	103	40	224	229
3	103	105	41	229	234
4	105	108	42	234	239
5	108	110	43	239	244
6	110	112	44	244	249
7	112	115	45	249	254
8	115	117	46	254	260
9	117	120	47	260	265
10	120	122	48	265	271
11	122	125	49	271	276
12	125	127	50	276	282
13	127	130	51	282	288
14	130	133	52	288	294
15	133	135	53	294	301
16	135	138	54	301	307
17	138	141	55	307	314
18	141	144	56	314	320
19	144	147	57	320	327
20	147	150	58	327	334
21	150	154	59	334	341
22	154	157	60	341	348
23	157	160	61	348	356
24	160	164	62	356	363
25	164	167	63	363	371
26	167	171	64	371	379
27	171	174	65	379	387
28	174	178	66	387	395
29	178	182	67	395	403
30	182	186	68	403	412
31	186	190	69	412	421
32	190	194	70	421	430
33	194	198	71	430	439
34	198	202	72	439	448
35	202	206	73	448	458
36	206	210	74	458	467
37	210	215	75	467	477
38	215	220	–	–	–

Figure S2 shows exemplary transmittance spectra of a filter with $M = 64$ and $N = 75$. The spectra are taken from Fig. 2d of the main text. For normal incidence ($\theta_i = 0\,^\circ$), all incident radiation is transmitted through the filter. We call this configuration the OFF-state of the filter. Conversely, for an incident angle $\theta^* = 23\,^\circ$, the filter is ON. In this case, the incident radiation from 0.26 to 2.6 μm (solar spectrum delimited by the vertical lines) is reflected and beyond this wavelength all radiation is transmitted.

COMPLEMENTARY POWER CALCULATIONS

Energy balance for the radiator and filter

Figure S3 shows diagrams of the power inputs and outputs considered in the energy balance of the (a) radiator and the (b) filter. Yellow arrows indicate inputs (positive terms) and blue arrows indicate outputs (negative terms). This represents schematically Equations (1) and (2) of the main text. P_{dev} contributes in both cases, because is the power radiated by the combined radiator-filter system, i.e., it depends on the optical properties of both the filter and the radiator. P_{exc} accounts only for the radiative transfer between the bottom surface of the filter and the radiator (in steady-state, this term is always zero, as shown in Fig. 3c of the main text).

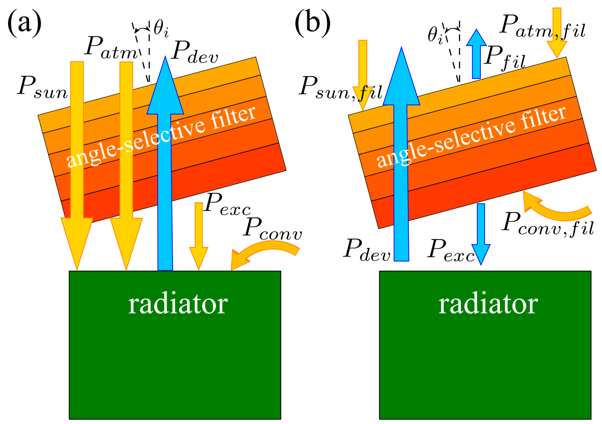

FIG. S3. (a) Diagram of input and output powers involved in the energy balance of the radiator. (b)Diagram of input and output powers involved in the energy balance of the filter.

Power expressions for the filter

$P_{sun,fil}$ is the input power per unit area that the filter absorbs from the sun. Accordingly:

$$P_{sun,fil} = \int_0^\infty I_{AM1.5}(\lambda)\epsilon_{fil}(\lambda)\mathrm{d}\lambda, \qquad \text{(S2)}$$

where $I_{AM1.5}(\lambda)$ is the spectral irradiance of the Sun.[2] $\epsilon_{fil}(\lambda)$ is the spectral emissivity of the angle-selective filter. It is computed using Kirchhoff's law ($\epsilon_{fil}(\lambda) = A_{fil}(\lambda) = 1 - R_{fil}(\lambda) - T_{fil}(\lambda)$). The emissivity of the (non-absorbing) filter is almost zero. Consequently, $P_{sun,fil} \approx 0$ for all incidence angles.

The input power radiated from the atmosphere to the filter is

$$P_{atm,fil}(T_{amb}) = \pi \int_0^\infty \int_0^{\pi/2} I_{BB}(T_{amb},\lambda)\epsilon_{fil}(\lambda)\epsilon_{atm}(\lambda,\theta)\sin 2\theta \mathrm{d}\theta\mathrm{d}\lambda, \qquad \text{(S3)}$$

where $I_{BB}(T,\lambda)$ is the black-body spectral radiance at temperature T. In equation (S3), $T = T_{amb}$. The emissivity of the atmosphere is computed as $\epsilon_{atm}(\lambda,\theta) =$

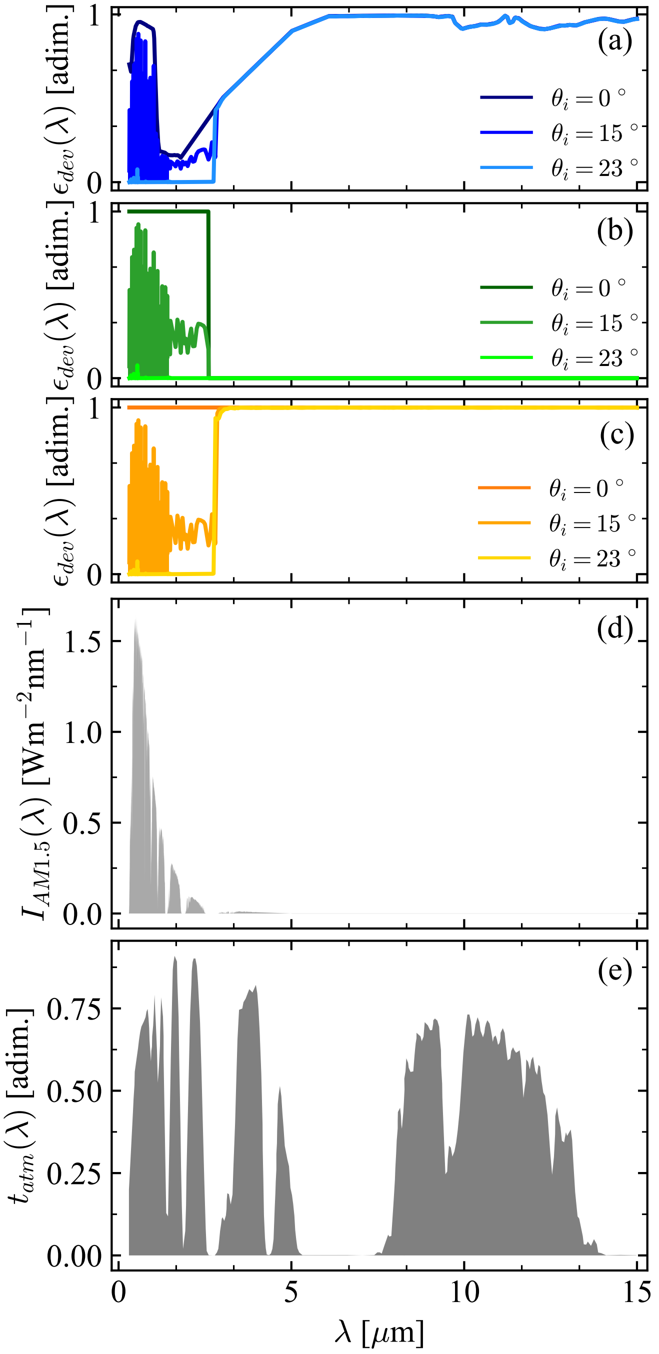

FIG. S4. Spectral emissivity of the device (radiator in presence of the filter) at three different incidence angles when the radiator is: (a) a photonic solar absorber covered by a transparent thermal blackbody, (b) an ideal solar absorber and (c) an ideal total absorber. (d) Spectral irradiance of the sun and (e) transmittance spectrum of the atmosphere.

$1 - [1 - t_{atm}(\lambda, 0\,^{o})]^{1/\cos\theta}$ and $t_{atm}(\lambda, 0\,^{o})$ is the transmittance spectrum of the atmosphere at zenit.[3] Because the emissivity of the filter is almost zero, it is expected a small contribution from the atmosphere to the heating of the filter.

The convective loss at both surfaces of the filter is

$$P_{conv,fil}(T, T_{fil}) = h_{conv}\left(T_{amb} - T_{fil}\right) + h_{conv}\left(\frac{T+T_{fil}}{2} - T_{fil}\right), \tag{S4}$$

where h_{conv} is the heat transfer coefficient due to convection to the surrounding air. The air gap temperature is considered as the mean value between the temperature of the radiator T and the temperature of the filter T_{fil}.

The exchanged power by radiation between the radiator surface and the bottom surface of the filter can be computed as:

$$P_{exc}(T, T_{fil}) = \pi \int_0^\infty \frac{\left[I_{BB}(T_{fil},\lambda) - I_{BB}(T,\lambda)\right]\epsilon_{fil}(\lambda)\epsilon_{rad}(\lambda)}{\epsilon_{fil}(\lambda) + \epsilon_{rad}(\lambda) - \epsilon_{fil}(\lambda)\epsilon_{rad}(\lambda)} \mathrm{d}\lambda, \tag{S5}$$

where $\epsilon_{rad}(\lambda)$ and $\epsilon_{fil}(\lambda)$ are the spectral emissivities of the radiator and the filter, respectively. Under steady-state conditions, the exchange power between the radiator and filter goes to zero.

The cooling power of the device, i.e., the radiator in presence of the filter, is given by:

$$P_{dev}(T) = \pi \int_0^\infty I_{BB}(T, \lambda)\epsilon_{dev}(\lambda)\mathrm{d}\lambda. \tag{S6}$$

The power radiated by the upper surface of the filter can be computed as:

$$P_{fil}(T_{fil}) = \pi \int_0^\infty I_{BB}(T_{fil}, \lambda)\epsilon_{fil}(\lambda)\mathrm{d}\lambda. \tag{S7}$$

This power is practically zero, because the emissivity of the filter is almost zero. The filter does not present radiative cooling characteristics by itself.

Figure S4a shows exemplary spectra of the emissivity of the device $\epsilon_{dev}(\lambda)$ at three different incidence angles. For normal incidence ($\theta_i = 0\,^\circ$), the emissivity of the device coincides with the one of the radiator surface. In this case, a solar absorber covered by a visibly transparent nocturnal radiator is considered.[4] The dark blue line corresponds to its emission spectrum. Furthermore, another two extreme cases of radiators are considered: an ideal solar absorber, whose emissivity is one over the entire solar spectrum and zero at any other wavelength (including the sky-window), as shown by the dark-green spectrum in Fig. S4b. Likewise, an ideal total absorber, whose emissivity is one over the entire spectrum (also within the sky-window), as shown in Fig. S4c in dark-orange color. In all cases, the emissivity within the solar spectrum becomes almost zero for $\theta_i = 23\,^\circ$. The spectral irradiance of the sun $I_{AM1.5}(\lambda)$ is shown in Fig. S4d and Fig. S4e shows the transmittance spectrum $t_{atm}(\lambda)$ of the atmosphere.

Ambient temperature performance

Table S2 shows numerical values of the temperature of a nocturnal radiator (T) and the temperature of a solar filter (T_{fil}) as the incidence angle is changed, the filter

TABLE S2. Steady-state temperatures and power contributions per unit area for a radiator (solar absorber covered by a visibly transparent nocturnal radiator) and filter at ambient temperature $T_{amb} = 298.3$K.

θ_i [deg]	T [K]	T_{fil} [K]	P_{atm} [Wm^{-2}]	P_{dev} [Wm^{-2}]	P_{conv} [Wm^{-2}]	P_{sun} [Wm^{-2}]	P_{exc} [Wm^{-2}]	P_{fil} [Wm^{-2}]	$P_{atm,fil}$ [Wm^{-2}]	$P_{conv,fil}$ [Wm^{-2}]	$P_{sun,fil}$ [Wm^{-2}]
0	351.56	270.12	104.34	551.15	-325.73	772.55	-1.20E-14	2.96E-15	7.64E-16	551.15	1.91E-15
5	350.34	270.48	104.34	541.96	-319.43	757.05	7.06E-04	-2.91E-04	-1.08E-04	541.96	-2.01E-04
10	334.82	274.31	104.33	433.98	-242.05	571.69	-6.00E-05	1.83E-05	-1.00E-05	433.98	-4.21E-05
15	299.24	278.18	104.32	245.24	-84.24	225.16	1.09E-05	-1.32E-05	3.91E-06	245.24	5.41E-06
18	280.75	277.87	104.30	174.96	-11.51	82.17	-4.68E-07	1.59E-06	-3.39E-06	174.96	1.02E-04
23	269.96	277.07	104.24	141.38	28.44	8.71	2.93E-06	1.59E-05	1.22E-05	141.38	-4.26E-04
25	269.78	277.06	104.20	140.78	29.13	7.45	2.00E-06	1.47E-05	1.26E-05	140.78	-2.23E-04
28	269.78	277.07	104.08	140.64	29.17	7.39	1.45E-06	2.58E-05	1.17E-05	140.64	2.80E-06
30	269.79	277.08	104.01	140.57	29.18	7.38	-3.52E-06	-4.07E-05	-1.07E-05	140.57	-1.44E-06
31	269.79	277.09	103.96	140.51	29.18	7.38	-5.45E-07	-2.01E-06	3.85E-06	140.51	2.64E-06
35	269.83	277.13	103.64	140.19	29.20	7.35	3.89E-06	3.69E-05	1.00E-05	140.19	9.97E-06
40	269.90	277.20	103.08	139.60	29.21	7.31	-9.40E-07	2.79E-06	4.08E-06	139.60	-2.92E-06

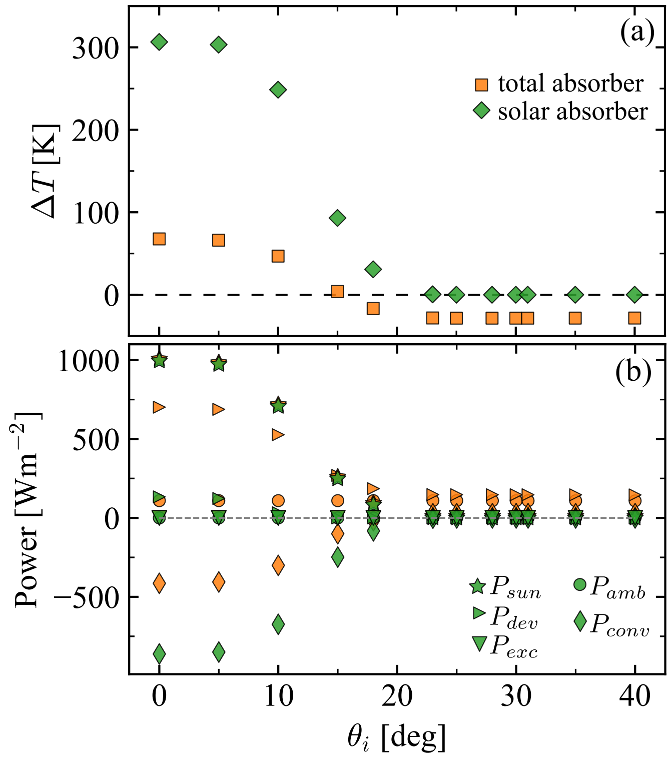

FIG. S5. (a) Temperature difference ($\Delta T = T - T_{amb}$) between the radiator surface and the ambient as a function of the incidence angle. (b) Computed power contributions per unit area as a function of the incidence angle (see Eq. (1) of main text). Ambient temperature $T_{amb} = 298.3$ K is considered in these calculations.

is tilted. This radiator-filter device is the one studied in the main text. The surrounding atmosphere is at temperature $T_{amb} = 298.3$ K. All power contributions per unit area considered in equations (1) and (2) of the main text are also listed.

The heating power from the sun P_{sun} to the radiator drops two orders of magnitude when the incidence angle with respect to the filter is equal or larger than 23 °. Consequently, the nocturnal radiator can be heated up by solar radiation ($\theta_i < 23\,°$) or not heated up ($\theta_i \geq 23\,°$).

The negative sign of the convective power P_{conv} from the radiator surface to the air gap for $\theta_i < 23\,°$ is because for those incidence angles, the steady-state temperature of the radiator is larger than that of the filter ($T > T_{fil}$), i.e., the radiator surface is losing heat by convection to the air gap. This behavior is expected, due to the fact that the radiator used is a nocturnal radiative cooler surface and solar absorber. Hence, its emissivity is high in the solar spectrum.[4] Consequently, it is heated up when the solar radiation is not properly blocked by the filter. On the other hand, for $\theta \geq 23\,°$, the radiator surface is always at lower temperature than the one of the filter ($T < T_{fil}$). In this circumstances, the convective power from the radiator to the filter is positive, i.e., the air gap is losing heat by convection to the radiator surface.

The total convective power $P_{conv,fil}$ on the filter is always positive because, the difference between the ambient temperature and the filter temperature is always larger than the difference between the air gap mean temperature and the filter temperature.

In the case of replacing the radiator by an ideal solar absorber, its surface can be maintained at ambient temperature ($\Delta T \approx 0$) only for optimum operation conditions of the angle-selective solar filter ($\theta_i \geq 23\,°$), as shown in Fig. S5a by green diamond markers. For lower incidence angles, part of the solar radiation reaches the ideal solar absorber and its surface temperature increases accordingly. For normal incidence, all solar radiation is absorbed by the ideal solar absorber and its temperature rises around 306 K above the ambient. All calculations were performed assuming steady-state conditions and $T_{amb} = 298.3$ K.

The incident solar radiation on the ideal solar absorber can be as high as around 1000 Wm^{-2} for normal incidence and drops down to around zero for $\theta \geq 23^{\circ}$ (see Fig. S5b, green star markers). The power radiated by the device is zero for $\theta \geq 23^{\circ}$, because the emissvity of the device within the sky-window is zero. Consequently, this ideal solar absorber does not present passive radiative cooling properties.

Finally, when replacing the radiator by an ideal total absorber, its surface can be cooled down to $\sim$ 28 K below the ambient, for optimum operation conditions of the angle-selective solar filter ($\theta_i \geq 23^{\circ}$), as shown in Fig. S5a by orange squared markers. For lower incidence angles, part of the solar radiation reaches the ideal total absorber and its surface temperature increases accordingly. For normal incidence, all solar radiation is absorbed by the ideal total absorber and its temperature rises around 68 K above the ambient. However, this temperature increase is 4.5 times lower than the corresponding temperature rise for the ideal solar absorber. This is because, the ideal total absorber also emits radiation through the sky-window as a cooling mechanism. All calculations were performed assuming steady-state conditions and $T_{amb} = 298.3$ K.

The incident solar radiation on the ideal total absorber can be as high as around 1000 Wm^{-2} for normal incidence and drops two orders of magnitude for $\theta \geq 23^{\circ}$ (see Fig. S5b, orange star markers). The net cooling power radiated by the device is $\sim$ 147 Wm^{-2} for $\theta \geq 23^{\circ}$, it is non zero because the emissvity of the device within the sky-window is one. Consequently, this ideal total absorber presents passive radiative cooling properties.

The sum of terms on the right hand side of Equation (1) in the main text is represented by the grey dashed line. $P_{total,rad} = 0$ for any incidence angle, as expected in the steady-state regime.

[1] P. Yeh, A. Yariv, and C.-S. Hong, J. Opt. Soc. Am. **67** (1977).

[2] ASTM G173-03, *Standard Tables for Reference Solar Spectral Irradiances: Direct Normal and Hemispherical on 37º Tilted Surface*, Standard (ASTM International, West Conshohocken, PA, 2012).

[3] A. Berk, G. P. Anderson, P. K. Acharya, L. S. Bernstein, L. Muratov, J. Lee, M. Fox, S. M. Adler-Golden, J. H. Chetwynd Jr., M. L. Hoke, R. B. Lockwood, J. A. Gardner, T. W. Cooley, C. C. Borel, P. E. Lewis, and E. P. Shettle, in *Algorithms and Technologies for Multispectral, Hyperspectral, and Ultraspectral Imagery XII*, Vol. 6233, edited by S. S. Shen and P. E. Lewis (SPIE, 2006).

[4] L. Zhu, A. P. Raman, and S. Fan, Proc. Natl. Acad. Sci. U. S. A. **112**, 12282 (2015).
